# Rapid detection of micronutrient components in infant formula milk powder using near-infrared spectroscopy

**DOI:** 10.3389/fnut.2023.1273374

**Published:** 2023-09-22

**Authors:** Shaoli Liu, Ting Lei, Guipu Li, Shuming Liu, Xiaojun Chu, Donghai Hao, Gongnian Xiao, Ayaz Ali Khan, Taqweem Ul Haq, Manal Y. Sameeh, Tariq Aziz, Manal Tashkandi, Guanghua He

**Affiliations:** ^1^School of Biological and Chemical Engineering, Zhejiang University of Science and Technology, Hangzhou, Zhejiang, China; ^2^Beingmate (Hangzhou) Food Research Institute Co., Ltd., Hangzhou, Zhejiang, China; ^3^Beingmate Dairy Co., Ltd., Anda, Heilongjiang, China; ^4^Department of Biotechnology, University of Malakand, Chakdara, Pakistan; ^5^Chemistry Department, Al-Leith University College, Umm Al-Qura University, Makkah, Saudi Arabia; ^6^Department of Agriculture, University of Ioannina, Ioannina, Greece; ^7^College of Science, Department of Biochemistry, University of Jeddah, Jeddah, Saudi Arabia

**Keywords:** infant formula milk powder, near-infrared spectroscopy, characteristic wavelengths, partial least squares regression, support vector regression

## Abstract

In order to achieve rapid detection of galactooligosaccharides (GOS), fructooligosaccharides (FOS), calcium (Ca), and vitamin C (Vc), four micronutrient components in infant formula milk powder, this study employed four methods, namely Standard Normal Variate (SNV), Multiplicative Scatter Correction (MSC), Normalization (Nor), and Savitzky–Golay Smoothing (SG), to preprocess the acquired original spectra of the milk powder. Then, the Competitive Adaptive Reweighted Sampling (CARS) algorithm and Random Frog (RF) algorithm were used to extract representative characteristic wavelengths. Furthermore, Partial Least Squares Regression (PLSR) and Support Vector Regression (SVR) models were established to predict the contents of GOS, FOS, Ca, and Vc in infant formula milk powder. The results indicated that after SNV preprocessing, the original spectra of GOS and FOS could effectively extract feature wavelengths using the CARS algorithm, leading to favorable predictive results through the CARS-SVR model. Similarly, after MSC preprocessing, the original spectra of Ca and Vc could efficiently extract feature wavelengths using the CARS algorithm, resulting in optimal predictive outcomes via the CARS-SVR model. This study provides insights for the realization of online nutritional component detection and optimization control in the production process of infant formula.

## 1. Introduction

Infant formula powder is highly favored by consumers due to its rich nutritional composition, including proteins, fats, carbohydrates, vitamins, minerals, and other essential nutrients for infant growth. It also offers advantages such as long shelf life and convenience in transportation. In the production process, accurate control of the content of proteins, fats, and carbohydrates is necessary. Additionally, precise monitoring of other micronutrients is crucial for ensuring the quality of the powder and is a key direction for future research in infant formula powder development (1–3). Infant formula manufacturing companies often fortify their formulas with oligosaccharides such as galacto-oligosaccharides (GOS) and fructo-oligosaccharides (FOS) to regulate the balance of infant gut microbiota, enhance immune function, and promote infant brain development. Nutrients like calcium (Ca) and vitamin C (Vc) are also added to enhance infant metabolism and support the generation of red blood cells and skeletal tissue. Therefore, the quantitative analysis of micronutrients in formula is crucial for quality control during the production process. Currently, conventional chemical detection methods are commonly used to determine the content of micro-nutrients such as GOS, FOS, Ca, and Vc in infant formula powder. However, these methods have drawbacks, including time-consuming sample preparation, complex procedures, and sample damage. The efficiency of conventional chemical methods is no longer sufficient to meet the requirements of accurate and intelligent control (4). Therefore, it has become an urgent need in the infant formula powder production industry to develop a rapid, efficient, and accurate online detection method for the content of micronutrients.

Near-infrared spectroscopy (NIRS) analysis, known for its simplicity, accuracy, rapidity, efficiency, and non-destructive nature, has been widely applied in various fields such as food (5–8), pharmaceuticals (9–11), and chemical engineering (12–14). It has also been utilized for rapid detection of milk powder and dairy products (15–17). The establishment of NIR fast detection model usually includes three processes: spectrum preprocessing by standard normal transform (SNV), feature wavelength extraction by competitive adaptive Reweighted sampling (CARS) and model establishment by partial least squares regression (PLSR). The accuracy of model prediction is also closely related to the algorithm used in the modeling process. Wu et al. (18) established a least square support vector regression (LSSVR) prediction model based on infrared spectroscopy, achieving the determination of milk powder brands and the detection of major nutritional components including proteins, fats, and carbohydrates. Asma et al. (15) developed a partial least square regression (PLSR) prediction model to predict the particle size, dispersibility, and bulk density of milk powder. Cattaneo and Holroyd (19) used near-infrared spectroscopy to establish a PLSR prediction model for detecting adulteration of melamine and microbial contamination in milk powder. Currently, most research focuses on brand determination, prediction of high-content nutrient levels, detection of physical properties and adulteration of milk powder (20, 21). However, due to the complex structures and low concentrations of micronutrients like GOS, there is limited literature on the rapid detection of GOS and FOS using NIRS analysis.

In this study, we aimed to establish a near-infrared quantitative model for micronutrients. Considering the complexity of infant formula powder composition, the variation in particle size, and the influence of external light radiation and noise during near-infrared spectroscopy scanning (22), this study aims to find the amount of GOS, FOS, Ca, and Vc micronutrients in infant formula powder by pre-processing the near-infrared spectra, extracting characteristic wavelengths, and establishing quantitative prediction models. This research will provide references for online detection and optimization control of nutritional components.

## 2. Materials and methods

### 2.1. Experimental materials

A total of 170 samples of infant formula powder were collected from an infant formula powder production company, including infant formula powder, larger infant formula powder, and toddler formula powder, with 120 samples containing GOS and 80 samples containing FOS. All samples contained Vc and Ca as nutritional components. After collection, the samples were stored in sealed bags to minimize the influence of external oxygen on the powder samples.

### 2.2. Spectral acquisition

Before collecting the spectra using a near-infrared spectrometer, the powder samples were kept at room temperature in the laboratory for a certain period to reduce measurement errors caused by temperature variations (23). The instrument was preheated for 30 min prior to measurement to prevent deviations from the true spectral characteristics (24). A Bruker MPA near-infrared spectrometer (Bruker Optics Inc., United States) was used to collect the near-infrared spectra of the samples. The spectral range was set from 800 nm to 2,500 nm, and the resolution was set at 4 cm^−1^ (25).

### 2.3. Chemical value determination

#### 2.3.1. Galactooligosaccharides content determination

The GOS content in the infant formula powder samples was determined using an enzymatic method (26). The GOS raw materials from the same batch were subjected to preprocessing analysis. The GOS content was measured using an ion chromatography-electrochemical pulse amperometric detector, which has high sensitivity. GOS raw materials usually contain other components such as lactose, glucose, and galactose. Based on the principle of consistent ratio of low-degree oligosaccharides in the raw materials and infant formula powder, a set of characteristic peaks for GOS was selected. The GOS content in the infant formula powder was indirectly determined using the same batch of raw material syrup as the reference. The content range of GOS in the test samples was determined to be 5–29 mg·kg^−1^ through chemical analysis.

#### 2.3.2. Fructooligosaccharides content determination

The FOS content in the milk powder samples was determined according to the national standard GB 5009.255–2016 “determination of fructosan in food.” The milk powder samples were extracted with hot water. The sucrose in the sample solution was hydrolyzed into glucose and fructose by sucrase. Glucose and fructose were then reduced to their corresponding sugar alcohols by sodium borohydride, and the excess sodium borohydride was neutralized with acetic acid. The fructosan in the sample solution were hydrolyzed into fructose and glucose by fructan hydrolase. The fructose content was determined using ion chromatography with pulsed amperometric detector. The content of fructosan was calculated based on conversion factors. The content range of FOS in the test samples was determined to be 4.4–26.6 mg·kg^−1^ through chemical analysis.

#### 2.3.3. Calcium content determination

The Ca content in the milk powder samples was determined according to the national standard GB 5009.92–2016 “determination of calcium in food.” Flame atomic absorption spectroscopy was used to measure the Ca content in the milk powder samples after digestion. Lanthanum solution was added as a releasing agent, and the absorbance values measured at 422.7 nm were proportional to the Ca concentration within a certain concentration range. The Ca content was quantitatively determined by comparing with a standard series. The content range of Ca in the test samples was determined to be 3.1–7.12 mg·kg^−1^ through chemical analysis.

#### 2.3.4. Vitamin C content determination

The Vc content in the milk powder samples was determined according to the national standard GB 5413.18–2010 “Determination of Vitamin C in Infant Food and Dairy Products.” Vc was oxidized to dehydroascorbic acid in the presence of activated carbon. It reacted with o-phenylenediamine to form a fluorescent substance, and the fluorescence intensity was measured using a fluorescence spectrophotometer. The fluorescence intensity was proportional to the concentration of Vc, and the content was quantified using an external standard method. The content range of Vc in the test samples was determined to be 0.54–1.82 mg·kg^−1^ through chemical analysis.

### 2.4. Spectral preprocessing

The infant formula powder samples were randomly divided into calibration and prediction sets in a 7: 3 ratio. The calibration set was used for model training, and the prediction set was used for model prediction. Four preprocessing methods, namely Standard Normal Variate (SNV), Multiplicative Scatter Correction (MSC), Normalization (Nor), and Savitzky–Golay smoothing (SG), were applied individually. The optimal preprocessing method was determined by establishing a Partial Least Squares (PLS) model.

### 2.5. Spectral feature wavelength extraction

#### 2.5.1. Competitive adaptive reweighted sampling algorithm

The CARS algorithm is a feature wavelength extraction method based on the theory of Darwinian evolution (27, 28). Absolute values of regression coefficients and the weights corresponding to each wavelength are calculated. It retains the wavelength points with the highest absolute weight coefficients and removes those with smaller weights. The feature wavelengths were determined based on the lowest Root Mean Square Error of Cross Validation (RMSECV). The parameters for the CARS algorithm were set as follows: maximum number of principal components = 10, number of cross-validations = 10, and number of Monte Carlo runs = 40.

#### 2.5.2. Random frog algorithm

The RF algorithm is an efficient method for selecting variables from high-dimensional data (29, 30). It calculates the probability of each wavelength being selected after N iterations and sorts them accordingly. The wavelengths with higher probabilities are selected for model building. To ensure convergence, the iteration parameter N was set to 10,000, and the number of selected feature wavelengths was set to 40.

### 2.6. Model establishment

#### 2.6.1. Partial least squares regression

The PLSR algorithm is used to establish prediction models (31, 32). The optimal number of latent variables for the PLSR model was also determined. After 10 rounds of training, the performance of the 10 models was evaluated, and the best hyperparameters were selected as the optimal number of latent variables for the PLS model.

#### 2.6.2. Support vector regression

The SVR algorithm uses a nonlinear kernel function to map low-dimensional input to a high-dimensional feature space and performs linear regression in the high-dimensional feature space (33, 34). It is suitable for handling problems with a small number of samples, nonlinearity, and high dimensionality. A Gaussian function was selected as the kernel function, and the values of the parameters c and g were set within the range of [−10, 10] with a step size of 0.5.

### 2.7. Model evaluation

The results of the models were evaluated using four indicators: related coefficient of calibration set (Rc), root mean square error of calibration set (RMSEC), related coefficient of prediction set (Rp), and root mean square error of prediction set (RMSEP) (35). A higher related coefficient indicates a closer prediction value to the true value and a stronger relationship between variables. A lower root mean square error indicates better model fitting ability.

## 3. Results and discussion

### 3.1. Spectral preprocessing

Four spectral preprocessing methods, namely SNV, MSC, Nor, and SG, were applied to the original spectra of the infant formula samples, and corresponding PLSR prediction models were established. The calibration set and prediction set were randomly divided in a 7: 3 ratio. The results of the PLSR models with different preprocessing methods are shown in Table 1. According to the evaluation criteria, it can be observed from the table that SNV preprocessing yielded better modeling results for GOS and FOS compared to the original spectra and the other three preprocessing methods. The PLSR prediction model for GOS achieved an Rc of 0.8093, RMSEC of 0.4289, Rp of 0.7592, and RMSEP of 0.4861. The PLSR prediction model for FOS yielded Rc of 0.8858, RMSEC of 0.1516, Rp of 0.8712, and RMSEP of 0.1948. Similarly, MSC preprocessing yielded better modeling results for Ca and Vc compared to the original spectra and the other three preprocessing methods. The PLSR prediction model for Ca achieved Rc of 0.8685, RMSEC of 0.0351, Rp of 0.8488, and RMSEP of 0.0426. The PLSR prediction model for Vc yielded an Rc of 0.6157, RMSEC of 0.0181, Rp of 0.5937, and RMSEP of 0.0247. Although there was an improvement in the model results after preprocessing, the overall improvement was not significant, especially for Vc. Therefore, further research is needed to explore feature wavelength extraction algorithms and other modeling methods to enhance the model performance.

**Table 1:** Modeling results of different pretreatment methods.

Nutrient composition	Pretreatment method	Rc	RMSEC	Rp	RMSEP
GOS	None	0.6724	0.5401	0.6695	0.5021
	**SNV**	**0.8093**	**0.4289**	**0.7592**	**0.4861**
	MSC	0.8045	0.4376	0.6727	0.4836
FOS	SG	0.7591	0.4847	0.577	0.4847
	Normaliz	0.7658	0.4568	0.6027	0.6474
	None	0.8025	0.1924	0.7095	0.3376
	**SNV**	**0.8858**	**0.1516**	**0.8712**	**0.1948**
	MSC	0.8394	0.1925	0.6533	0.2185
Ca	SG	0.8724	0.1571	0.7727	0.2578
	Normaliz	0.8684	0.1704	0.8162	0.2309
	None	0.8213	0.0397	0.8083	0.0393
	SNV	0.8678	0.0337	0.8233	0.0435
	**MSC**	**0.8685**	**0.0351**	**0.8488**	**0.0426**
Vc	SG	0.8464	0.0396	0.8121	0.0354
	Normaliz	0.8314	0.0356	0.8291	0.0436
	None	0.5347	0.0201	0.4055	0.0223
	SNV	0.5524	0.0175	0.5364	0.0245
	**MSC**	**0.6157**	**0.0181**	**0.5937**	**0.0247**
	SG	0.5602	0.0196	0.538	0.0211
	Normaliz	0.5179	0.0193	0.4833	0.0340

These are standard normal variate (SNV), multiplicative scatter correction (MSC).

**Figure 1:**
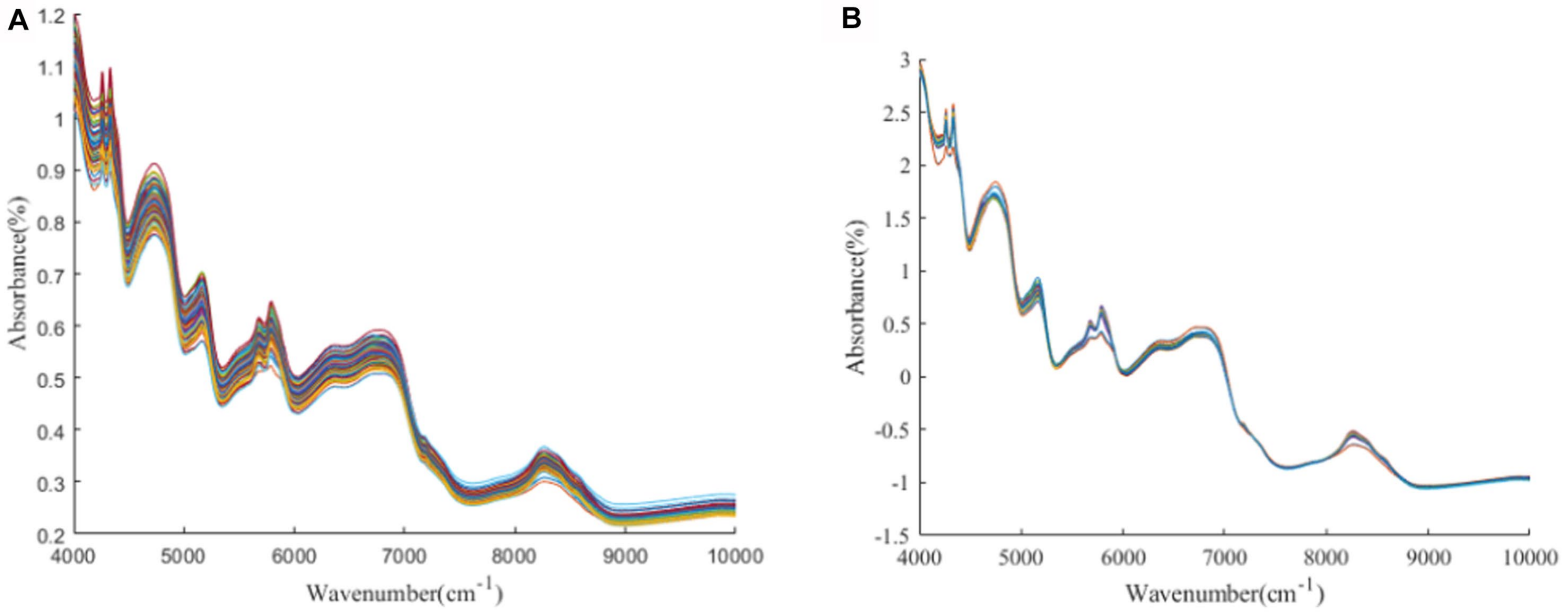
**(A, B)** GOS original spectrum and SNV pretreatment spectrum.

**Figure 2:**
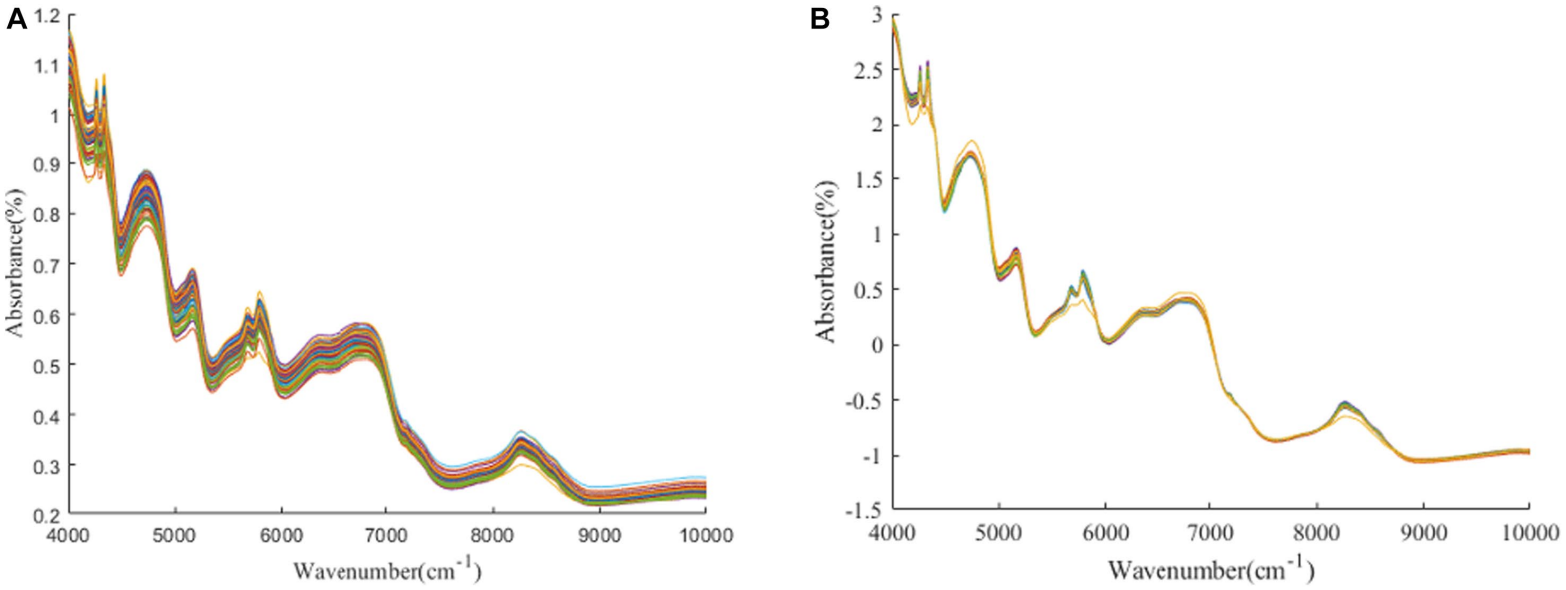
**(A, B)** FOS original spectrum and SNV pretreatment spectrum.

**Figure 3:**
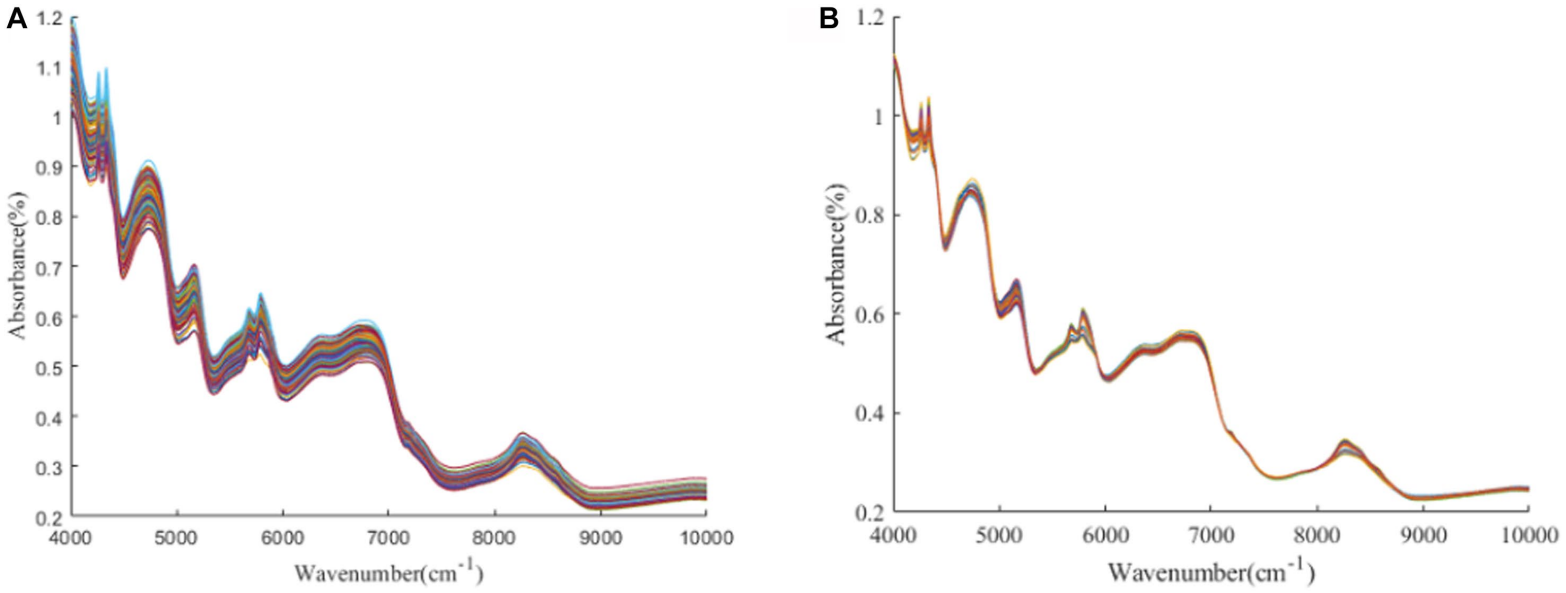
**(A, B)** Ca and Vc original spectrum and MSC pretreatment spectrum.

Figures 1–3 present the original spectral plots and the plots after optimal preprocessing for GOS, FOS, Ca, and Vc. Near-infrared spectra are usually influenced by the combination and overtone frequencies of hydrogen-containing groups such as O-H, N-H, and C-H (36). It can be observed from Figures 1A, 2A, 3A that the absorption peaks in the spectra of the experimental samples are generally consistent, with prominent characteristic peaks around 8,246 cm^−1^, 6,700 cm^−1^, 5,770 cm^−1^, 5,180 cm^−1^, and 4,748 cm^−1^. The preprocessed spectral plots are shown in Figures 1B, 2B, 3B, where MSC and SNV preprocessing methods effectively reduced the spectral interference caused by varying levels of external light scattering and enhanced the correlation between the spectra and the data, this is consistent with other research findings (37).

### 3.2. Feature wavelength selection

#### 3.2.1. Competitive adaptive reweighted sampling algorithm

**Figure 4:**
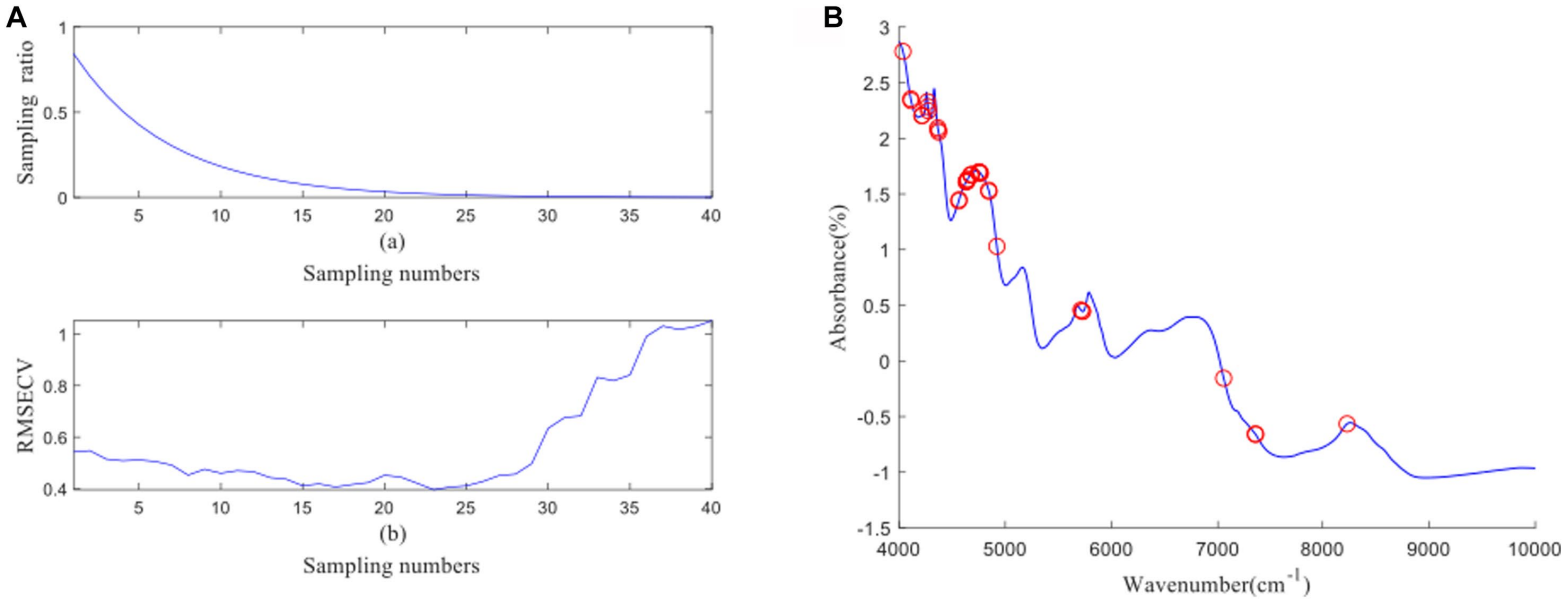
**(A, B)** GOS uses CARS algorithm to screen characteristic wavelength variable process and wavelength distribution.

**Figure 5:**
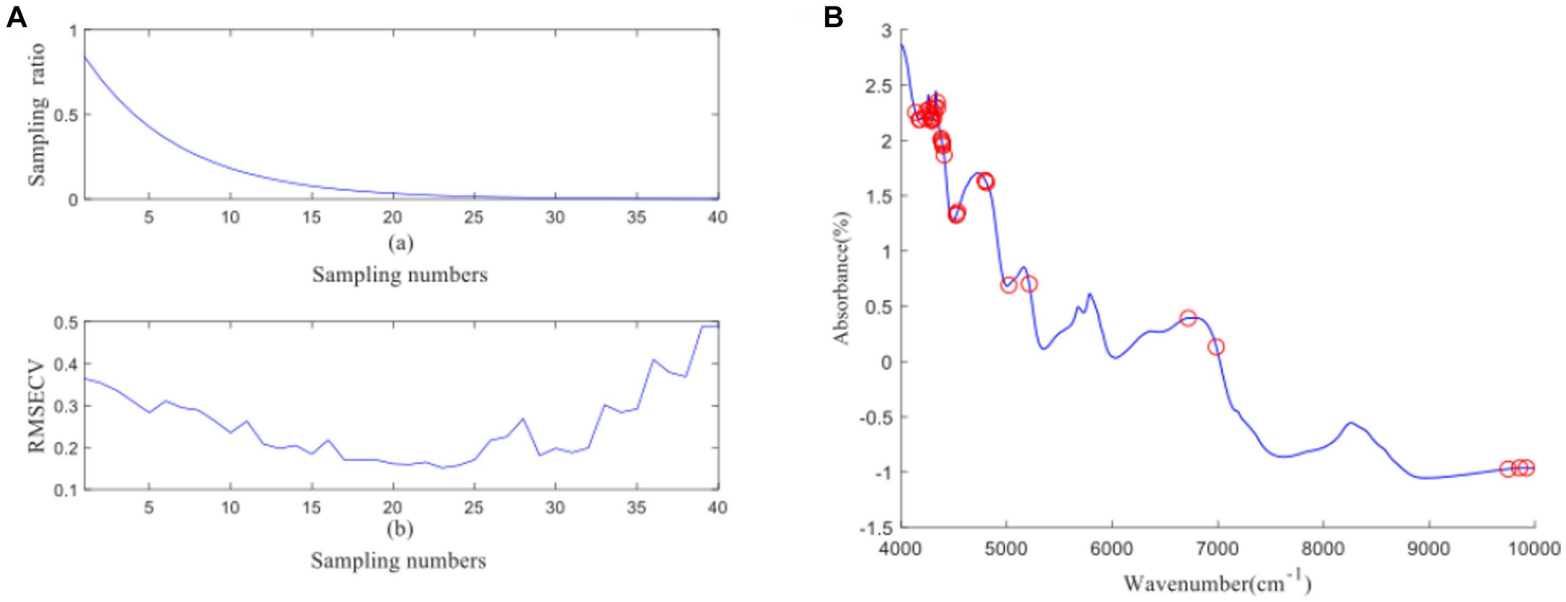
**(A, B)** FOS uses CARS algorithm to screen characteristic wavelength variable process and wavelength distribution.

**Figure 6:**
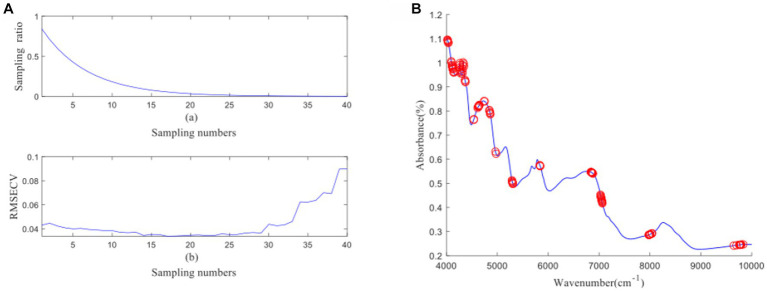
**(A, B)** Ca uses CARS algorithm to screen characteristic wavelength variable process and wavelength distribution.

**Figure 7:**
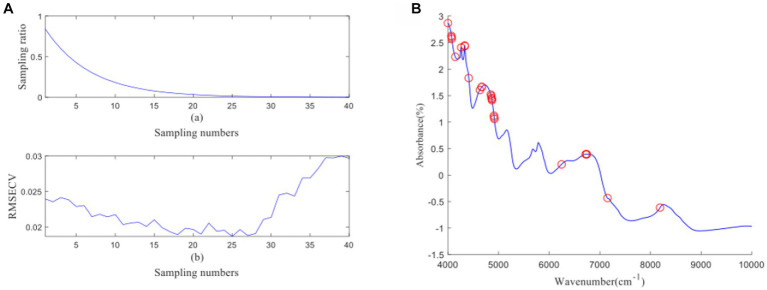
**(A, B)** Vc uses CARS algorithm to screen characteristic wavelength variable process and wavelength distribution.

Figure 4A depicts the process of feature wavelength extraction using CARS for GOS. From Figures 4Aa, it can be observed that as the number of samples increases, the sampling ratio of the variable subset starts to decrease and gradually stabilizes. Figures 4Ab shows that as the number of samples increases, the RMSECV of the eliminated unimportant wavelengths decreases slowly. However, it starts to increase when important wavelengths are eliminated. The lowest RMSECV is achieved when the Monte Carlo run reaches 23, resulting in the extraction of 26 feature wavelengths by CARS. The distribution of wavelengths is shown in Figure 4B. Figure 5A illustrates the process of feature wavelength extraction using CARS for FOS. The lowest RMSECV is achieved when the Monte Carlo run reaches 24, resulting in the extraction of 31 feature wavelengths. The distribution of wavelengths is shown in Figure 5B. Figure 6A shows the process of feature wavelength extraction using CARS for *Ca.* The lowest RMSECV is achieved when the Monte Carlo run reaches 17, resulting in the extraction of 101 feature wavelengths. The distribution of wavelengths is shown in Figure 6B. Figure 7A presents the process of feature wavelength extraction using CARS for Vc. The lowest RMSECV is achieved when the Monte Carlo run reaches 25, resulting in the extraction of 26 feature wavelengths. The distribution of wavelengths is shown in Figure 7B. Comparing the feature wavelengths selected by the CARS algorithm with the original full wavelengths, GOS, FOS, Ca, and Vc achieved reductions of 98.33, 98.01, 93.51, and 98.33%, respectively. This demonstrates that the CARS algorithm is capable of significantly reducing the number of wavelengths effectively (27).

#### 3.2.2. Random frog algorithm

**Figure 8:**
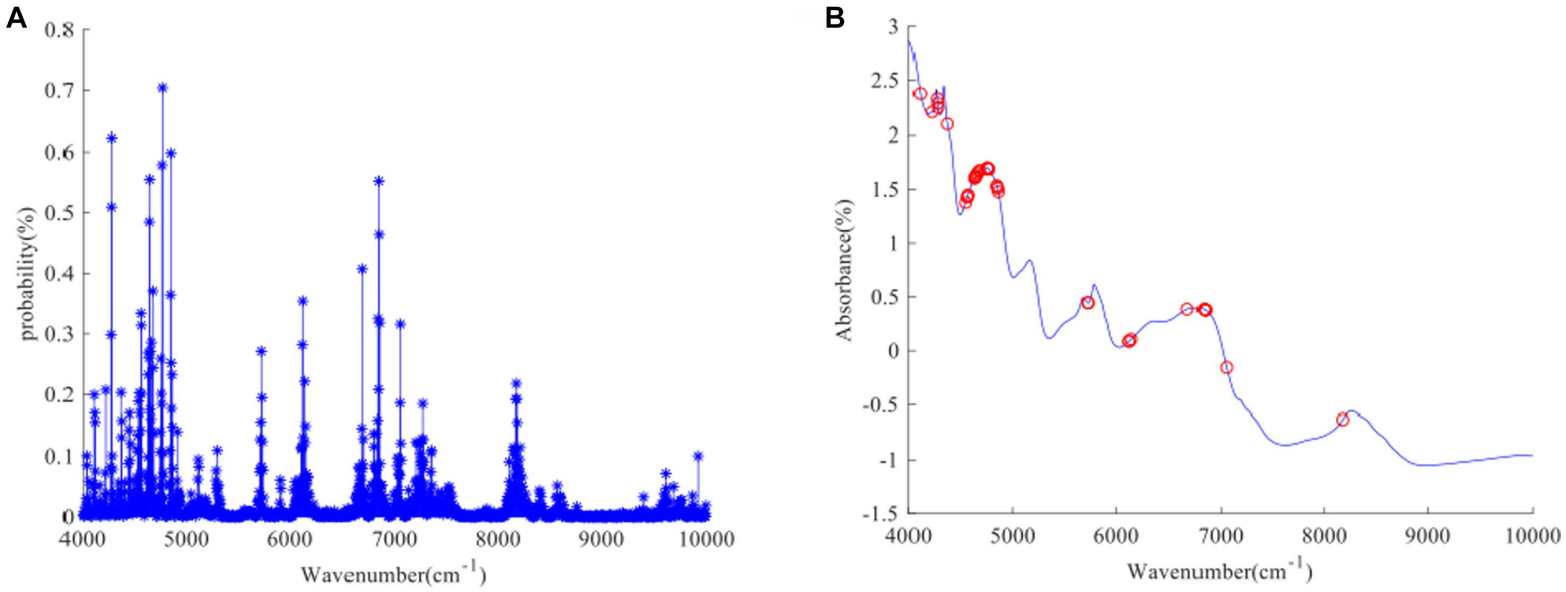
**(A, B)** GOS uses RF algorithm to screen characteristic wavelength process and characteristic wavelength distribution.

**Figure 9:**
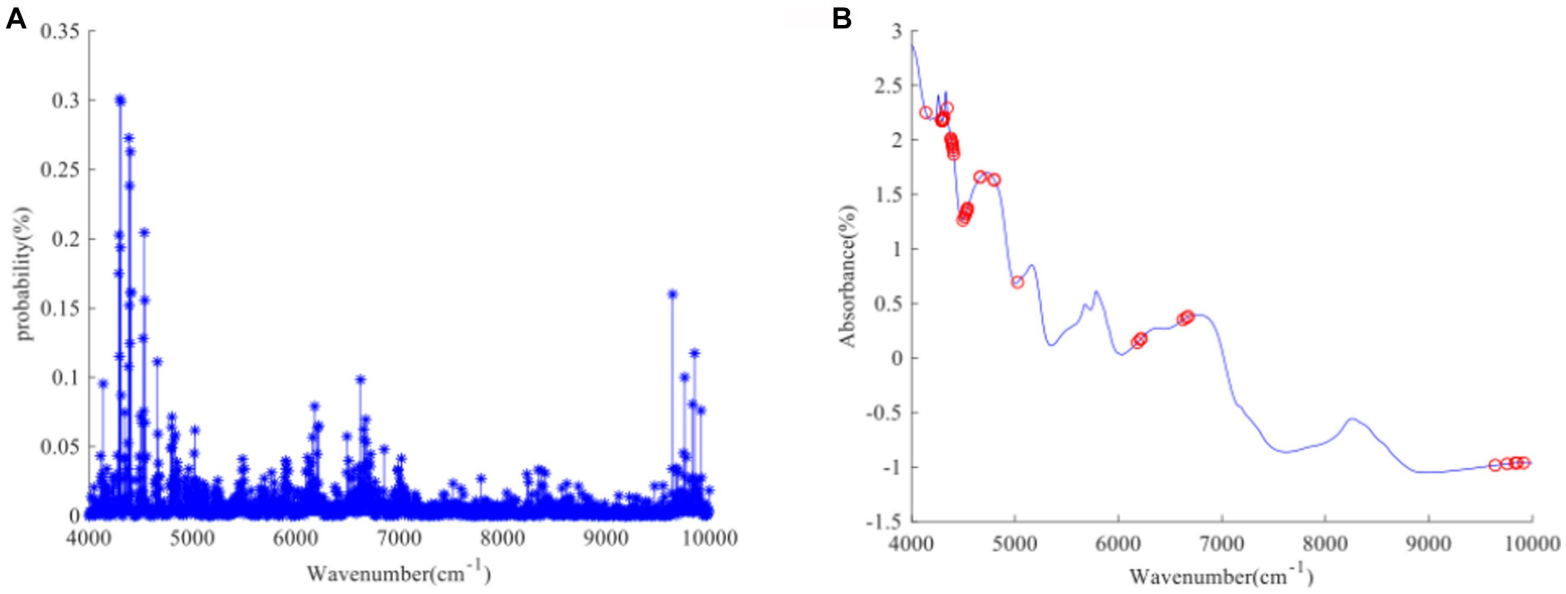
**(A, B)** FOS uses RF algorithm to screen characteristic wavelength process and characteristic wavelength distribution.

**Figure 10:**
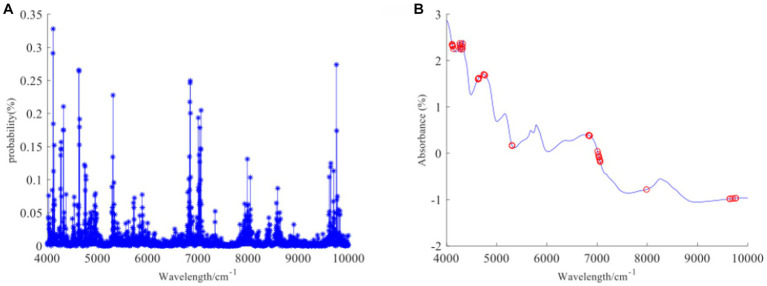
**(A, B)** Ca uses RF algorithm to screen characteristic wavelength process and characteristic wavelength distribution.

**Figure 11:**
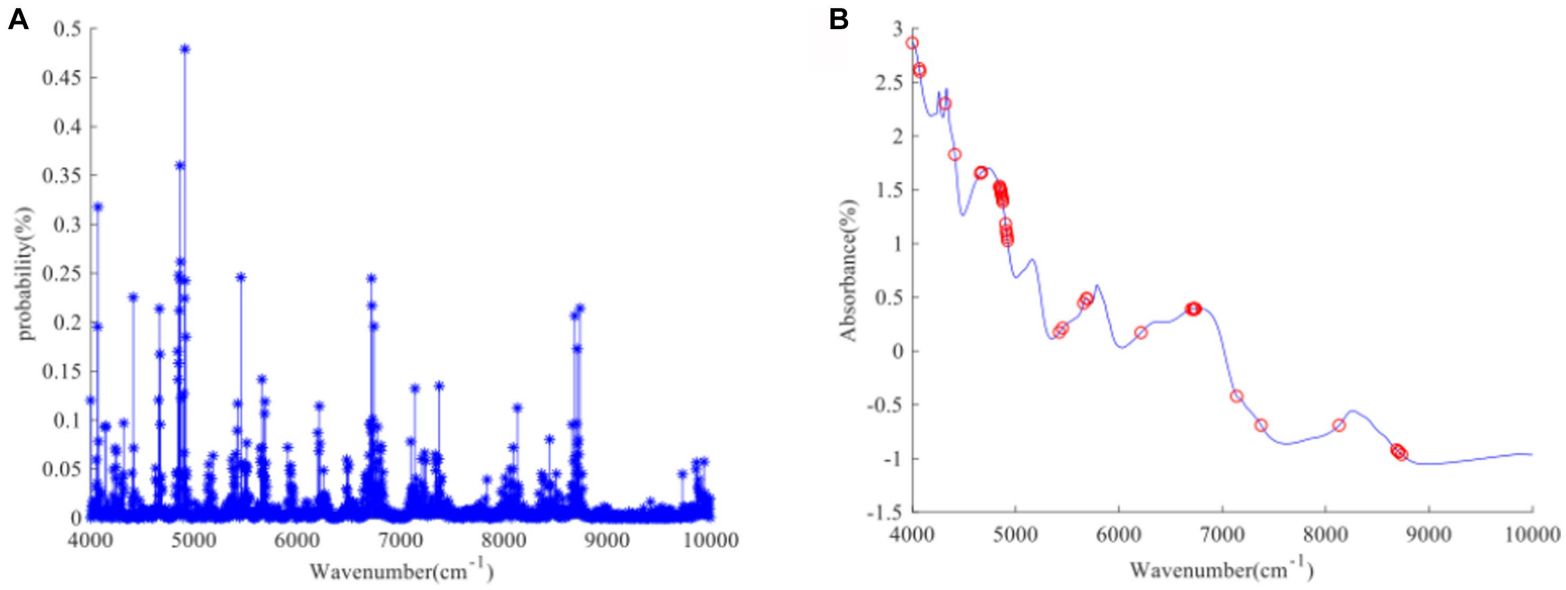
**(A, B)** Vc uses RF algorithm to screen characteristic wavelength process and characteristic wavelength distribution.

Figure 8A illustrates the probability of each wavelength being selected after 10,000 iterations during the process of feature wavelength extraction using the Random Frog (RF) algorithm for GOS. The x-axis represents the number of wavelengths, and the y-axis represents the probability of a wavelength being selected. From the figure, it can be observed that wavelengths near the absorption peaks at 4800 cm^−1^, 6,000 cm^−1^, 6,800 cm^−1^, and 8,100 cm^−1^ have a higher probability of being selected. Although wavelengths near other feature peaks are also selected, the probability of selection is relatively low. Ultimately, the top 40 wavelengths with the highest selection probabilities are chosen as feature wavelengths, as shown in Figure 8B. Figures 9A, 10A, 11A represent the probability of wavelength selection for FOS, Ca, and Vc, respectively. The distributions of the 40 selected feature wavelengths are shown in Figures 9B, 10B, 11B respectively. It can be observed that the selected feature wavelengths are distributed near the feature peaks. The feature wavelengths selected by the RF algorithm for GOS, FOS, Ca, and Vc achieved a reduction of 97.43% compared to the original full wavelengths. The RF algorithm has demonstrated effective feature wavelength extraction performance. The advantages of RF algorithm have also been verified in other literature, which is consistent with the conclusion of this study (29).

### 3.3. Model construction

#### 3.3.1. Partial least squares regression prediction models

PLSR prediction models for GOS, FOS, Ca, and Vc in infant formula milk were established based on feature wavelength extraction using the CARS and RF algorithms (Table 2). Compared to the full spectral range PLSR models, both the CARS-PLSR models and RF-PLSR models showed improved prediction performance for the four nutritional components. Considering the comprehensive evaluation of Rc and Rp, the prediction performance of the CARS-PLSR models was superior. The CARS-PLSR models for GOS, FOS, Ca, and Vc showed an increase in Rc by 0.0998, 0.0447, 0.0351, and 0.07, a decrease in RMSEC by 0.1146, 0.0406, 0.0055, and 0.001, an increase in Rp by 0.1362, 0.0521, 0.0440, and 0.0799, and a decrease in RMSEP by 0.1201, 0.0302, 0.0074, and 0.0071, respectively. This indicates that the CARS algorithm effectively reduces the interference of irrelevant spectral variables in modeling and improves the performance of the prediction models (28).

**Table 2:** Result of PLSR modeling.

Nutrient composition	Pretreatment method	Characteristic wavelength number	Rc	RMSEC	Rp	RMSEP
GOS	CARS-PLSR	26	0.9091	0.3143	0.8954	0.3660
	RF-PLSR	40	0.8878	0.3282	0.7612	0.6119
FOS	CARS-PLSR	31	0.9305	0.1110	0.9233	0.1646
	RF-PLSR	40	0.9308	0.1344	0.8824	0.1442
Ca	CARS-PLSR	101	0.9036	0.0296	0.8928	0.0352
	RF-PLSR	40	0.8728	0.0343	0.8528	0.0396
Vc	CARS-PLSR	26	0.6857	0.0171	0.6736	0.0176
	RF-PLSR	40	0.7016	0.0166	0.644	0.0219

#### 3.3.2. Support vector regression prediction models

Compared to the full spectral range PLS models, the CARS-SVR models and RF-SVR models showed significant improvements in the prediction performance for GOS, FOS, Ca, and Vc (Table 3). Considering the comprehensive evaluation of Rc and Rp, the prediction performance of the CARS-SVR models for all four nutritional components was superior to the RF-SVR models and CARS-PLS models. The CARS-SVR models showed an increase in Rc by 0.1480, 0.1104, 0.1187, and 0.3722, a decrease in RMSEC by 0.1267, 0.1063, 0.0193, and 0.0127, an increase in Rp by 0.1923, 0.0951, 0.0869, and 0.3502, and a decrease in RMSEP by 0.1857, 0.0616, 0.0007, and 0.0132 for GOS, FOS, Ca, and Vc, respectively.

**Table 3:** Result of SVR modeling.

Nutrient composition	Pretreatment method	Characteristic wavelength number	Rc	RMSEC	Rp	RMSEP
GOS	CARS-SVR	26	0.9573	0.3022	0.9515	0.3004
	RF-SVR	40	0.9566	0.2983	0.9273	0.4048
FOS	CARS-SVR	31	0.9962	0.0453	0.9663	0.1332
	RF-SVR	40	0.9859	0.0851	0.9547	0.1427
Ca	CARS-SVR	101	0.9872	0.0158	0.9357	0.0419
	RF-SVR	40	0.9506	0.0326	0.9353	0.0366
Vc	CARS-SVR	26	0.9879	0.0054	0.9439	0.0115
	RF-SVR	40	0.9892	0.0045	0.9218	0.0155

After establishing the SVR models based on feature wavelength extraction, it was found that the SVR models outperformed the PLSR models in both the calibration and prediction sets. This improvement can be attributed to the complex composition of infant formula milk powder, which contains multiple nutritional components. The interactions between different functional groups and absorption peaks of different categories contribute to the existence of complex nonlinear relationships between near-infrared spectroscopic data and the content of micronutrients in infant formula milk powder. The ability of PLSR to handle nonlinearity is significantly inferior to SVR. SVR, with its core utilization of nonlinear kernel functions, effectively enhances the correlation between spectroscopic data and the physicochemical content of the components (33, 34, 38).

**Figure 12:**
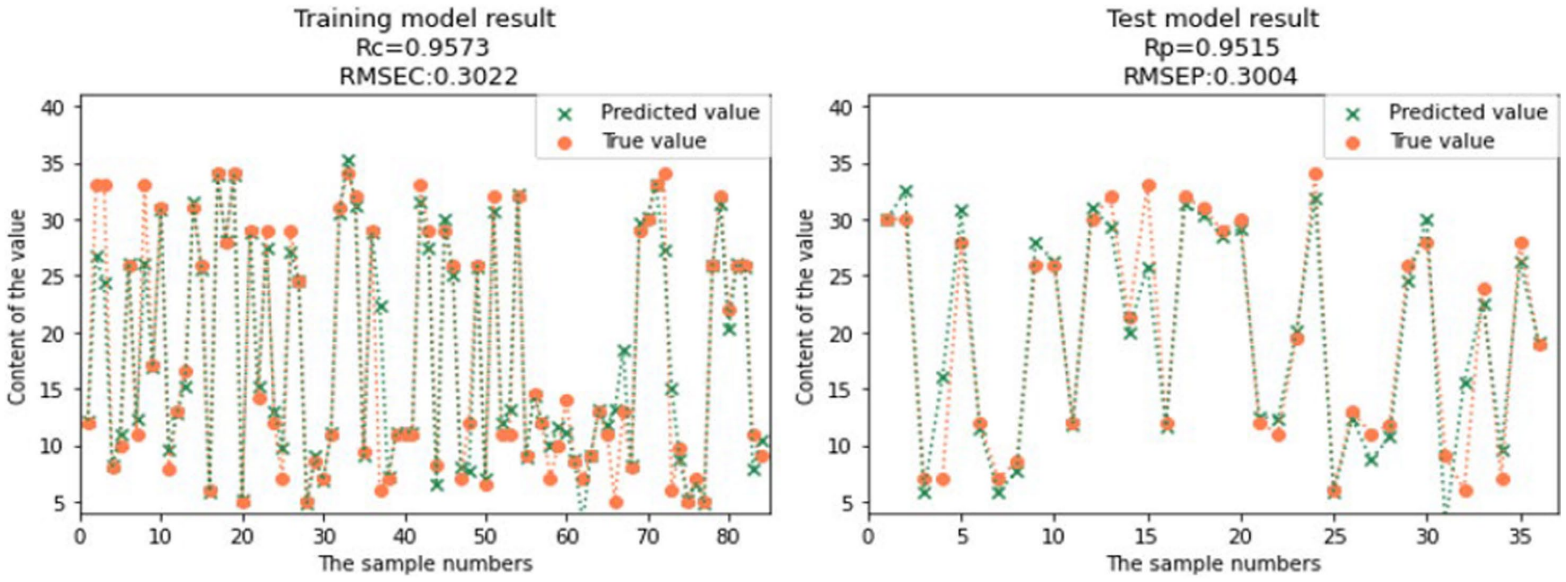
Comparison of GOS model predicted value and real value.

**Figure 13:**
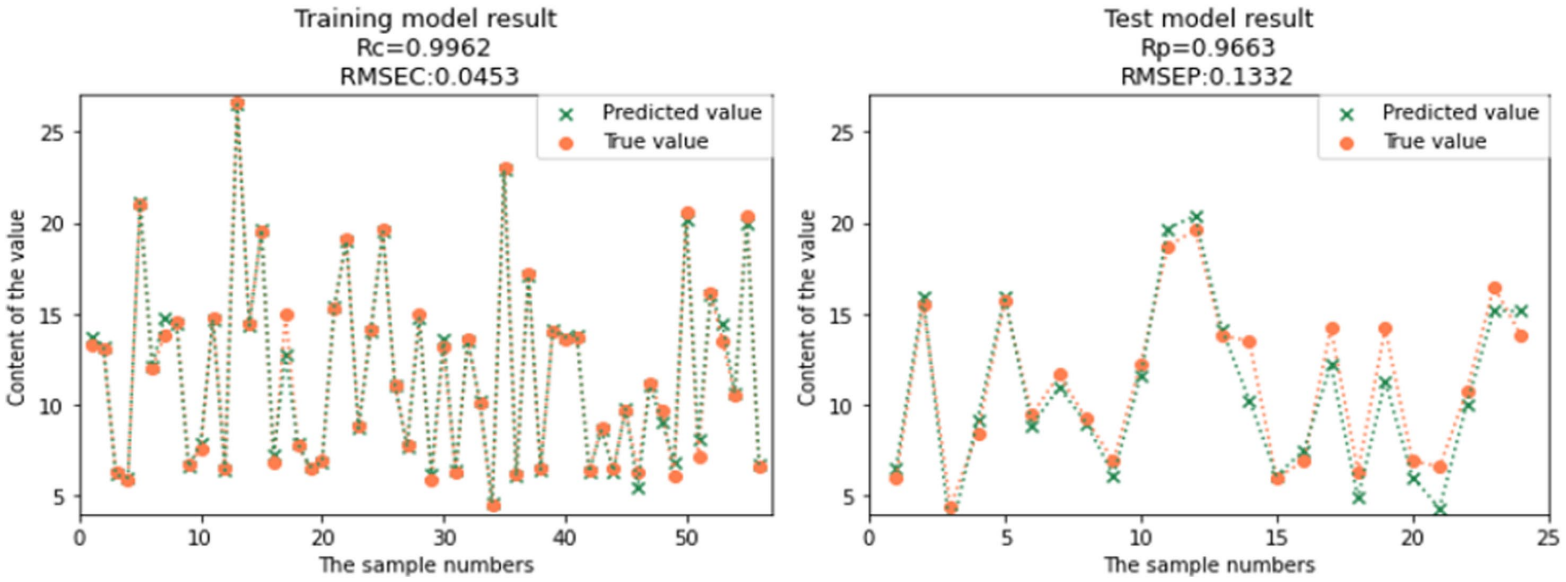
Comparison of FOS model predicted value and real value.

**Figure 14:**
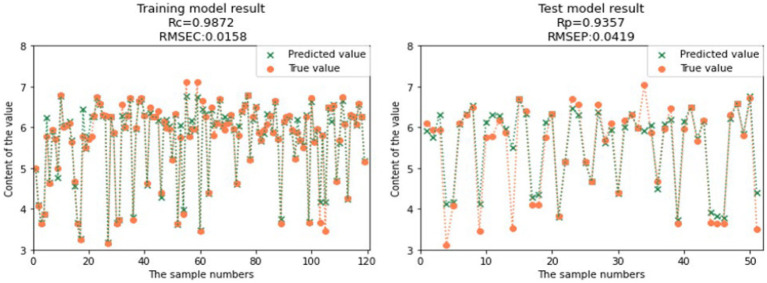
Comparison of Ca model predicted value and real value.

**Figure 15:**
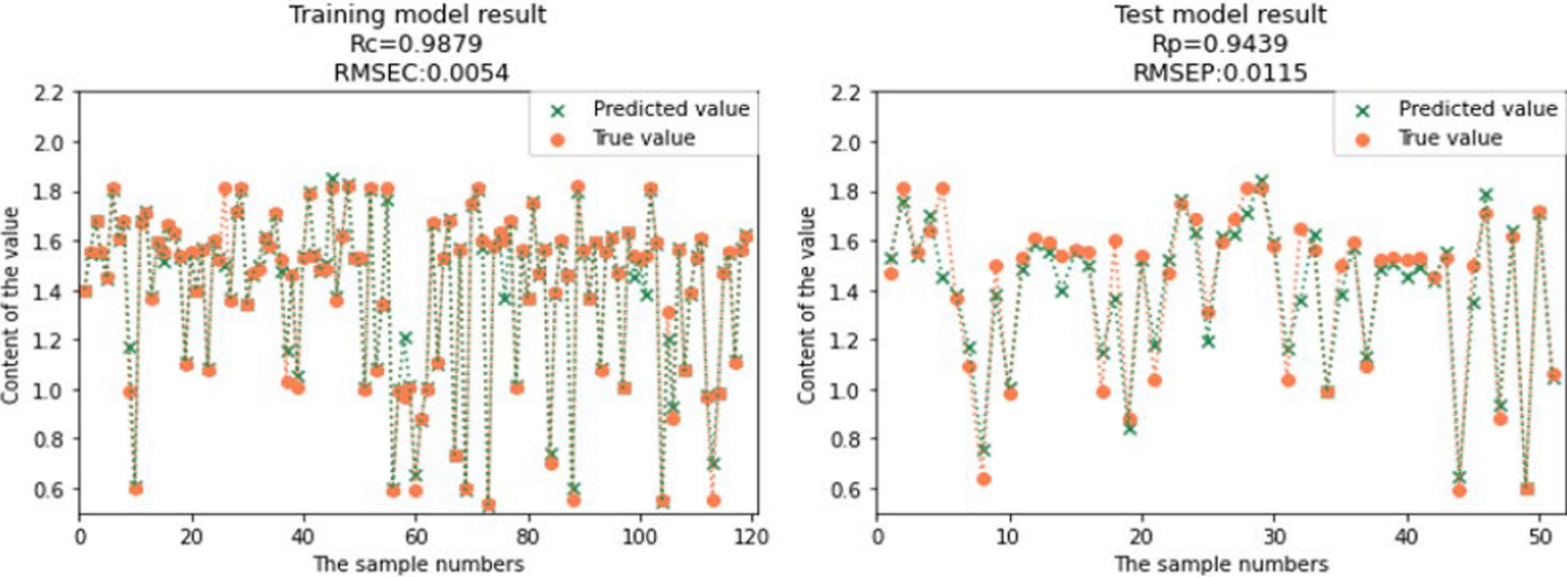
Comparison of Vc model predicted value and real value.

Therefore, utilizing feature wavelength extraction algorithms and establishing nonlinear SVR models is a more effective approach for the rapid detection of GOS, FOS, Ca, and Vc contents in infant formula milk powder. The comparison between the predicted values and true values of the CARS-SVR models for the four nutritional components is illustrated in Figures 12, 13, 14, 15. It can be observed from the figures that the deviation between the predicted and true values is low, indicating good calibration and prediction performance (39). To meet the requirements of online detection and optimization control in the milk powder production process, future research can focus on expanding the range of sample content, improving the applicability of the models, and investigating the feasibility of this method for rapid prediction in liquid milk powder ingredients (40–43). These efforts will provide valuable insights for online optimization control.

## 4. Conclusion

In this study, standard normal variate (SNV) preprocessing method was applied to preprocess the original spectra of GOS and FOS samples in infant formula milk powder, while multiplicative scatter correction (MSC) preprocessing method was applied to preprocess the original spectra of Ca and Vc samples. Feature wavelength extraction was performed using the CARS and RF algorithms, and PLSR and SVR models were established. Among them, the CARS-SVR model exhibited the best predictive performance, with Rc values of 0.9573, 0.9962, 0.9872, and 0.9879 for GOS, FOS, Ca, and Vc, respectively. The corresponding RMSEC values were 0.3022, 0.0453, 0.0158, and 0.0054, Rp values were 0.9515, 0.9663, 0.9357, and 0.9439, and RMSEP values were 0.3004, 0.1332, 0.0419, and 0.0115. This study provides a reference for the online detection and optimization control of nutritional components in the production process of infant formula milk powder.

## Data availability statement

The original contributions presented in the study are included in the article/supplementary material, further inquiries can be directed to the corresponding author.

## Author contributions

SaL and TL: conceptualization. GL: methodology. SuL: software. XC: validation. DH: formal analysis. GX: investigation. GH: resources, supervision, project administration, and funding acquisition. AK: data curation. TA: writing–original draft preparation. TH and MT: writing–review and editing. MS: visualization. All authors contributed to the article and approved the submitted version.
